# Prediction of malignant transformation and recurrence of oral epithelial dysplasia using architectural and cytological feature specific prognostic models

**DOI:** 10.1038/s41379-022-01067-x

**Published:** 2022-03-31

**Authors:** Hanya Mahmood, Mike Bradburn, Nasir Rajpoot, Nadim Mohammed Islam, Omar Kujan, Syed Ali Khurram

**Affiliations:** 1 Academic Unit of Oral & Maxillofacial Surgery, School of Clinical Dentistry, University of Sheffield, 19 Claremont Crescent, Sheffield, S10 2TA UK; 2 Clinical Trials Research Unit, School of Health and Related Research, University of Sheffield, Sheffield, UK; 3 Tissue Image Analytics Centre, Department of Computer Science, University of Warwick, Coventry, UK; 4 Department of Oral & Maxillofacial Diagnostic Sciences, College of Dentistry, University of Florida, Gainesville, FL USA; 5 Oral Diagnostic and Surgical Sciences Division, UWA Dental School, The University of Western Australia, Perth, WA Australia; 6 Unit of Oral & Maxillofacial Pathology, School of Clinical Dentistry, University of Sheffield, Sheffield, UK

**Keywords:** Oral cancer, Prognostic markers

## Abstract

Oral epithelial dysplasia (OED) is a precursor state usually preceding oral squamous cell carcinoma (OSCC). Histological grading is the current gold standard for OED prognostication but is subjective and variable with unreliable outcome prediction. We explore if individual OED histological features can be used to develop and evaluate prognostic models for malignant transformation and recurrence prediction. Digitised tissue slides for a cohort of 109 OED cases were reviewed by three expert pathologists, where the prevalence and agreement of architectural and cytological histological features was assessed and association with clinical outcomes analysed using Cox proportional hazards regression and Kaplan–Meier curves. Within the cohort, the most prevalent features were basal cell hyperplasia (72%) and irregular surface keratin (60%), and least common were verrucous surface (26%), loss of epithelial cohesion (30%), lymphocytic band and dyskeratosis (34%). Several features were significant for transformation (*p* < 0.036) and recurrence (*p* < 0.015) including bulbous rete pegs, hyperchromatism, loss of epithelial cohesion, loss of stratification, suprabasal mitoses and nuclear pleomorphism. This led us to propose two prognostic scoring systems including a ‘6-point model’ using the six features showing a greater statistical association with transformation and recurrence (bulbous rete pegs, hyperchromatism, loss of epithelial cohesion, loss of stratification, suprabasal mitoses, nuclear pleomorphism) and a ‘two-point model’ using the two features with highest inter-pathologist agreement (loss of epithelial cohesion and bulbous rete pegs). Both the ‘six point’ and ‘two point’ models showed good predictive ability (AUROC ≥ 0.774 for transformation and 0.726 for recurrence) with further improvement when age, gender and histological grade were added. These results demonstrate a correlation between individual OED histological features and prognosis for the first time. The proposed models have the potential to simplify OED grading and aid patient management. Validation on larger multicentre cohorts with prospective analysis is needed to establish their usefulness in clinical practice.

## Introduction

Oral epithelial dysplasia (OED) is a chronic, progressive precursor epithelial disorder of the oral mucosa, characterised by abnormal maturation and stratification of the surface epithelium^1^. It is associated with a statistically increased risk of progression to oral squamous cell carcinoma (OSCC) which is among the topmost common cancers worldwide and has an increasing incidence and worsening prognosis^2,3^. Clinically, OED most commonly presents as a white patch/plaque (leukoplakia) with up to 50% of biopsied lesions showing dysplasia^4^ and malignant transformation rate of 9.5% [99% CI 5.9–14.00%] or 1.56% per year^5^. OED can also be seen in other oral potentially malignant disorders (OPMD), a group of lesions and conditions characterised by an increased risk of malignant transformation, including oral submucous fibrosis, actinic keratosis, erythroplakia and erythroleukoplakia^6,7^. The presence of OED in these disorders increases their risk of malignant transformation^8^.

At present, there are no biological or molecular markers proven to be prognostically significant (or in routine diagnostic use) for OED^4^. Histological grading remains the gold standard for predicting malignancy risk and is used to inform patient treatment and prognosis^9^. Over the years, OED grading systems have substantially evolved, and the current World Health Organisation (WHO) classification (2017) grades dysplasia based on the presence of sixteen different histological features^10^. The ‘severity’ of these features, both in terms of frequency and location in the epithelium, are used to classify lesions into ‘low’, ‘moderate’ and ‘high’ grades, representing an increasing risk for malignant transformation^9^. A recent meta-analysis showed moderate/severe OED to be associated with a greater risk of malignant transformation compared to mild OED with an odds ratio of 2.4 (99% CI 1.5–3.8)^5^. However, it remains unclear which lesions will progress, and which will recur, as the mechanisms for OED progression are poorly understood^9^. Furthermore, the histological features can individually be considered relatively non-specific, and some (or all) of the features may be seen in different grades of dysplasia, of which some lesions will transform, and others will not (irrespective of grade).

In addition to these issues, there are a number of other problems related to the current grading system^11^. Firstly, there is substantial subjectivity in histological interpretation between pathologists, which can result in wide inter- and intra-observer variability, with potential for an incorrect grade being assigned^12^. This variability can arise since individual features are ill-defined, and this is further complicated by division of the epithelium into ‘thirds’ which can be challenging. Secondly, grading does not reliably predict prognosis which means that lower grade lesions may progress to OSCC whereas higher grade lesions may remain static^4,10^. Thirdly, several of the established histologic features can also be seen in reactive lesions, such as the margins of ulcers or candida infections. It is accepted that a complex interaction exists between a combination of features including histological atypia, progressive molecular changes and chromosomal derangements to trigger cancer development, but the individual importance of these features in OED progression is not well established^13,14^.

More recently, an alternative binary grading system (low/high grade) has been proposed^15^. This system grades dysplasia based on the overall number of cytological and architectural changes observed, and several studies have shown its improved reproducibility, inter-observer agreement and clinical utility as compared to the WHO system^15,16^. Despite these improvements though, neither systems consider the importance of individual histological features, or specify which of the features (in isolation or combination) are of greatest relevance for transformation and recurrence. Some older studies have compared OPMDs that did not transform to lesions that did^17^, and others have linked certain histology features to a higher transformation risk^18^. However, conclusions from these studies should be treated with caution due to weaknesses in the proposed methodologies.

The aims of this study are twofold: first, to conduct a detailed histological assessment (and inter-observer agreement) of individual OED features to identify which were most prevalent and associated with a higher risk of malignant transformation and recurrence; second, to develop and propose feature-specific prognostic models for OED outcome prediction. To the best of our knowledge, this is the first study to explore histological feature-specific prognostic prediction of OED.

## Materials/subjects and methods

### Case selection, tissue preparation and conversion to digital images

A retrospective sample of sequential OED cases were retrieved between 2008 and 2013 from the Oral and Maxillofacial Pathology archive at the School of Clinical Dentistry (Sheffield, UK) using a local digital database (ethical approval: 18/WM/0335). To confirm cases which had progressed to OSCC at the same clinical site, a regional head and neck cancer (HNC) electronic records system was accessed which is a repository for HNC cases within South Yorkshire. Newly stained 4 µm Haematoxylin and Eosin (H&E) sections of the selected cases were obtained from formalin fixed paraffin embedded blocks and a digital slide scanner (Aperio CS2, Milton Keynes, UK) was used to obtain whole slide images (WSI) at x40 magnification.

### Inclusion and exclusion criteria

The principal inclusion criteria were varying grades of OED retrieved from the Sheffield Oral and Maxillofacial Pathology archive with sufficient available tissue and availability of minimum five-year follow-up data. Where multiple biopsies had been taken over a period of follow-up, only the initial biopsy was selected for the study. The unit of Oral and Maxillofacial Pathology at Sheffield is a regional and national referral centre which receives referrals from a wide geographical area, however, following a confirmed tissue diagnosis any necessary treatment is provided by a local core Oral and Maxillofacial team and therefore cases treated outside this unit were by default excluded in this study. Additionally, cases were excluded if there was insufficient tissue for histological analysis, incomplete minimum follow up data or histological evidence of positive tissue margins on the subsequent excision (to avoid any bias in the recurrence data). The H&E slide and clinical records for all selected cases were reviewed by two authors (HM, SAK) to ensure the inclusion criteria was met.

### Clinical data collection

Minimum five-year follow-up data was obtained from clinical notes and biopsy forms by HM. Data collection included patient demographics/characteristics (age, gender, intraoral site), histological OED grade and two main clinical outcomes of interest (time to transformation and recurrence). Transformation was defined as a dysplastic lesion which had progressed to OSCC at the same clinical site and within the follow-up period, and recurrence was defined as a dysplastic lesion which occurred again in the same clinical site following active treatment (i.e. surgical excision or laser treatment) within the follow-up period. All data was recorded by HM in a structured proforma using Microsoft Excel (2016) in an anonymised-linked format.

### Histological evaluation and examiners

Three experienced oral and maxillofacial pathologists (NMI, OK, SAK) working in different international centres performed independent histological examination of the OED cohort. All pathologists were provided access to the WSIs via a cloud-based system. Each WSI was labelled with an anonymous-linked number, and all pathologists were blinded to the original diagnosis and clinical outcomes. The examiners were asked to independently assess the cases and identify which histological features amongst the WHO criteria were present and informed the diagnosis. They were also encouraged to specify any additional histological features which were considered important in influencing their diagnosis.

To determine which OED features were most prevalent, the examiners were asked to provide a binary score to record the presence (or absence) of individual features; a score of 1 was given if the feature was abundantly visible (and influenced diagnosis), and a score of 0 if the feature was absent or rare/focal. The topmost common histological OED features (as per consensus scoring) were further explored to determine feature-specific observer agreement and prognostic significance. To minimise examiner bias, no formal calibration exercises were attempted, although there was an informal discussion between the examiners to discuss their approach to this task. For consistency and to prevent double counting of similar appearing histological features, the pathologists agreed on general definitions for individual WHO features (as well as other commonly presenting features). For example, basal cell hyperplasia was considered if crowding/proliferation involved 1–2 layers of basal cells, whereas loss of epithelial stratification was considered if there was a disturbance in the organised ‘stratified’ layers of the epithelium and the layers were haphazardly organised or difficult to separate.

Finally, the original OED histological grades were independently reviewed by HM and where necessary, an updated grade was assigned. A standardised score sheet was designed in Microsoft Excel (2016) to record all examiner scoring and aid systematic analysis. All participating pathologists were clinical-academic pathologists with long-standing experience in the diagnosis of OED and OSCC.

### Statistical evaluation

Statistical analyses were conducted using the Stata Statistical Software^19^ (Version 17, 2021). The prevalence of OED features was calculated overall and for each examiner. Observer agreement was summarised as the percentage of patients for whom all three examiners agreed, and by two chance-corrected measures (Cohen’s Kappa and Gwet’s AC), where a value of 1 denotes perfect agreement and 0 relates to no agreement beyond chance alone.

Univariate associations between pathological features and clinical outcomes (transformation and recurrence) were visualised by Kaplan–Meier curves and analysed using a Cox proportional hazards regression model with Efron’s correction for tied times. Thereafter, two prognostic models were developed in which the outcome of interest was event (transformation and recurrence) at any time. The prognostic performance of the two models were compared against each other as well as against patient/clinical characteristics (age, gender, intraoral site) and histological OED grade alone by generating the area under the receiver-operator characteristic curve (AUROC). All statistical tests were two-tailed and *p* < 0.05 were considered statistically significant.

## Results

### Characteristics of the study cohort

151 previously diagnosed cases of OED were retrieved during the study period, of which 42 were excluded due to either insufficient tissue availability or incomplete minimum five-year clinical follow up data. Amongst the patient cohort, 67 (61%) were male and 42 (39%) were female with a median age of 67 years (IQR 57–77). Breakdown based on intraoral site were as follows: tongue 44 (40%), floor of mouth 23 (21%), buccal mucosa 17 (16%), gingivae 7 (6%), soft palate 6 (6%), hard palate 6 (6%) and lower lip 6 (6%). The clinical records showed that 34 (31%) of OED lesions were clinically monitored, 70 (64%) were surgically excised and 5 (5%) were treated with laser.

### Prevalence and agreement of OED features

The final study cohort (Table 1) comprised 109 OED cases which were blindly re-evaluated to confirm 34 (31%) mild, 48 (44%) moderate and 27 (25%) severe dysplasia cases. Binary grading of these cases showed 73 (67%) to be low grade and 36 (33%) as high-grade lesions. Table 2 summarises the prevalence and observer agreement for the twelve most prominent OED features that were observed as per consensus scoring. The most common features were basal cell hyperplasia (72%) and irregular surface keratin (60%). The latter feature refers to any irregularity of the keratin layer, including a corrugated, shaggy or desquamative appearance. This feature was included since all pathologists highlighted it as a prominent feature in certain cases, and at present it is not on the list of WHO criteria. The least common were verrucous surface morphology (26%), loss of epithelial cohesion (30%), lymphocytic band (34%) and dyskeratosis (34%). All other features ranged between 36% and 57%.

**Table 1:** Characteristics of the study cohort.

Characteristic	Number (%) or median (IQR)
Age	67 (57–77)
Gender	
Female	42 (39%)
Male	67 (61%)
WHO grade	
Mild	34 (31%)
Moderate	48 (44%)
Severe	27 (25%)
Binary grade	
Low	73 (67%)
High	36 (33%)
Site of disease	
Tongue	44 (40%)
Floor of mouth	23 (21%)
Buccal mucosa	17 (16%)
Gingivae	7 (6%)
Hard palate	6 (6%)
Lower lip	6 (6%)
Soft palate	6 (6%)

**Table 2:** Observer agreement for OED feature analysis.

Prominent OED Features	Overall prevalence*	Complete agreement	Cohen’s Kappa	Gwet’s AC1
Basal cell hyperplasia	236 (72%)	63 (58%)	0.30	0.53
Bulbous/drop shaped rete pegs	187 (57%)	72 (66%)	0.54	0.56
Dyskeratosis	110 (34%)	68 (62%)	0.44	0.55
Hyperchromatism	176 (54%)	53 (49%)	0.32	0.32
Irregular surface keratin	196 (60%)	68 (62%)	0.48	0.52
Loss of epithelial cohesion	98 (30%)	80 (73%)	0.58	0.69
Loss of stratification	138 (42%)	61 (56%)	0.41	0.43
Suprabasal mitoses	148 (45%)	54 (50%)	0.34	0.34
Nuclear pleomorphism	118 (36%)	62 (57%)	0.38	0.47
Abrupt orthokeratosis	174 (53%)	81 (74%)	0.66	0.66
Lymphocytic band	112 (34%)	79 (72%)	0.60	0.67
Verrucous surface	85 (26%)	92 (84%)	0.73	0.83

*Denominator for overall prevalence is the number of assessments (327; 109 patients each with 3 assessments). Complete agreement is the percentage of patients (out of 109) where all three assessors agreed.

Verrucous surface morphology had the highest agreement between pathologists (Kappa = 0.73, Gwet’s AC1 = 0.83). Gwet’s AC1 measurements were comparable for abrupt orthokeratosis (0.66), lymphocytic band (0.67) and loss of epithelial cohesion (0.69). Agreement for all other features was typically modest, with the worst agreement for hyperchromatism (Kappa and Gwet’s AC1 both = 0.32) and suprabasal mitoses (Kappa and Gwet’s AC1 both = 0.34) for which all three pathologists agreed for approximately half the patients.

### OED feature-specific incidence of transformation and recurrence

Table 3 summarises feature-specific incidence for transformation and recurrence. Overall, 20 (18%) OED lesions transformed, and 27 (25%) lesions recurred following treatment. A higher incidence of transformation was seen when bulbous/drop shaped rete pegs (30%), loss of epithelial cohesion (35%), loss of stratification (34%) and nuclear pleomorphism (32%) were observed. The incidence of recurrence was also higher related to these same four features, as well as suprabasal mitoses (37%) and nuclear pleomorphism (41%).

**Table 3:** Incidence of transformation and recurrence by OED feature.

Overall	Transformation 20 (18%)		Recurrence 27 (25%)	
Prominent OED Features	Positive	Negative	Positive	Negative
Basal cell hyperplasia	15 (18%)	5 (20%)	19 (23%)	8 (32%)
Bulbous/drop shaped rete pegs	18 (30%)	2 (4%)	20 (33%)	7 (14%)
Dyskeratosis	8 (24%)	12 (16%)	12 (36%)	15 (20%)
Hyperchromatism	15 (26%)	5 (10%)	20 (34%)	7 (14%)
Irregular surface keratin	10 (15%)	10 (24%)	16 (24%)	11 (26%)
Loss of epithelial cohesion	11 (35%)	9 (12%)	14 (45%)	13 (17%)
Loss of stratification	15 (34%)	5 (8%)	19 (43%)	8 (12%)
Suprabasal mitoses	14 (27%)	6 (10%)	19 (37%)	8 (14%)
Nuclear pleomorphism	13 (32%)	7 (10%)	17 (41%)	10 (15%)
Abrupt orthokeratosis	10 (17%)	10 (20%)	14 (23%)	13 (27%)
Lymphocytic band	9 (25%)	11 (15%)	12 (33%)	15 (21%)
Verrucous surface	6 (20%)	14 (18%)	8 (27%)	19 (24%)

For each feature, a consensus definition was used whereby the feature was assumed to be present if 2/3 observers rated it as being prominent, otherwise it was assumed absent.

### Feature-specific correlation to clinical outcomes

Table 4 summarises the hazard ratios and *p* values of individual OED features for their time to the two clinical outcomes of interest (malignant transformation and recurrence). Six features were associated with a greater rate of transformation: bulbous/drop shaped rete pegs (*p* = 0.005) hyperchromatism (*p* = 0.036), loss of epithelial cohesion (*p* = 0.003), loss of stratification (*p* = 0.001), suprabasal mitoses (*p* = 0.022) and nuclear pleomorphism (*p* = 0.005).

**Table 4:** Hazard ratios and *p* values of individual OED features for their time to malignant transformation and recurrence.

Prominent OED features	Transformation		Recurrence	
	Hazard ratio	*p* value	Hazard ratio	*p* value
Basal cell hyperplasia	0.88 (95% CI 0.32, 2.42)	0.806	0.65 (95% CI 0.29, 1.49)	0.310
Bulbous rete pegs	8.27 (95% CI 1.92, 35.68)	0.005*	2.52 (95% CI 1.06, 5.96)	0.036*
Dyskeratosis	1.68 (95% CI 0.69, 4.11)	0.257	2.20 (95% CI 1.03, 4.70)	0.042*
Hyperchromatism	2.96 (95% CI 1.08, 8.15)	0.036*	2.90 (95% CI 1.23, 6.86)	0.015*
Irregular surface keratin	0.62 (95% CI 0.26, 1.49)	0.286	0.92 (95% CI 0.43, 1.99)	0.841
Loss of epithelial cohesion	3.78 (95% CI 1.57, 9.14)	0.003*	3.50 (95% CI 1.64, 7.46)	0.001*
Loss of stratification	5.35 (95% CI 1.94, 14.73)	0.001*	4.50 (95% CI 1.97, 10.30)	0.000*
Suprabasal mitoses	3.06 (95% CI 1.17, 7.96)	0.022*	3.17 (95% CI 1.39, 7.24)	0.006*
Nuclear pleomorphism	3.74 (95% CI 1.49, 9.38)	0.005*	3.45 (95% CI 1.58, 7.54)	0.002*
Abrupt orthokeratosis	0.78 (95% CI 0.32, 1.87)	0.572	0.85 (95% CI 0.40, 1.81)	0.680
Lymphocytic band	1.80 (95% CI 0.75, 4.35)	0.191	1.74 (95% CI 0.82, 3.73)	0.151
Verrucous surface	1.09 (95% CI 0.42, 2.85)	0.855	1.11 (95% CI 0.49, 2.53)	0.807

*Denotes a statistically significant finding.

These same six features (bulbous/drop shaped rete pegs *p* = 0.036, hyperchromatism *p* = 0.015, loss of epithelial cohesion *p* = 0.001, loss of stratification *p* < 0.001, suprabasal mitoses *p* = 0.006, nuclear pleomorphism *p* = 0.002), in addition to dyskeratosis (*p* = 0.042), were also positively associated with recurrence.

### Proposed prognostic models for OED

Two prognostic models were explored to assess the potential for reliably predicting clinical outcomes of OED. In all cases, the number of covariates was minimised to limit the impact of overfitting.

#### Prognostic model 1: Six-point scoring system

The first scoring system allocated one point for the presence of each of the six OED features which were associated with a greater incidence of transformation and recurrence (bulbous/drop shaped rete pegs, hyperchromatism, loss of epithelial cohesion, loss of stratification, suprabasal mitoses, nuclear pleomorphism). Since the hazard ratios for these features (Table 4) are reasonably similar, each feature is allocated equal weight.

**Fig. 1:**
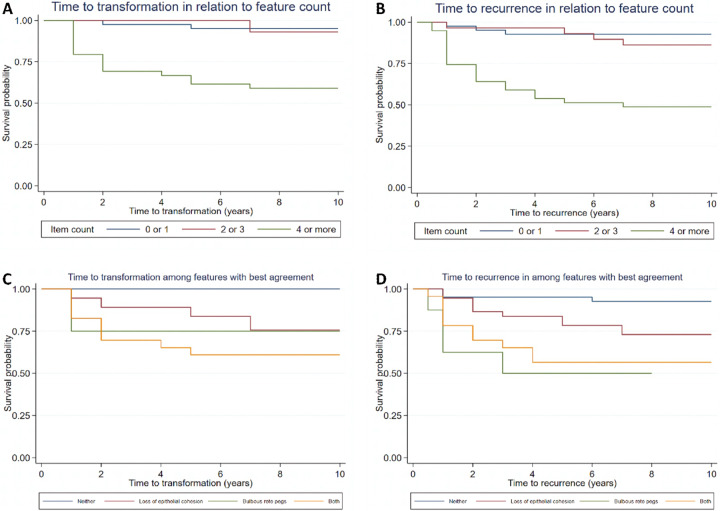
Kaplan–Meier curves for time to transformation and recurrence for feature count based on the six-point scoring system (**A**, **B**) and the two-point scoring system (**C**, **D**).

Figure 1A and B (see Supplementary Material) present the Kaplan–Meier survival curves for time to transformation and time to recurrence in relation to the number of features present using the six-point scoring model. The predicted transformation rate at 2 years is estimated at 2% (95% CI 0–16%) for 0–1-point scoring, 0% for 2–3-point scoring and 31% (95% CI 19–48%) for 4–6-point scoring. At 5 years, these figures increase to 5% (95% CI 1–18%) for 0–1-point scoring and 38% (95% CI 25–55%) for 4–6-point scoring; there is no change in the rate for 2–3-point scoring (0%). For recurrence of OED, the respective predicted rates at two and five years were shown to be: 5% (95% CI 1–18%) and 7% (95% CI 2–21%) for 0–1-points; 3% (95% CI 0–22%) and 7% (95% CI 2–25%) for 2–3-points; 36% (95% CI 23–53%) and 49% (95% CI 34–65%) for 4–6 points. The lower recurrence and transformation rate seen for 2–3-point scoring compared to 0–1 points is unexpected but is likely to be related to the much lower number of cases in the 2–3 point category compared to the others. Validation on a more balanced larger cohort would be useful to determine the significance of these findings.

**Fig. 2:**
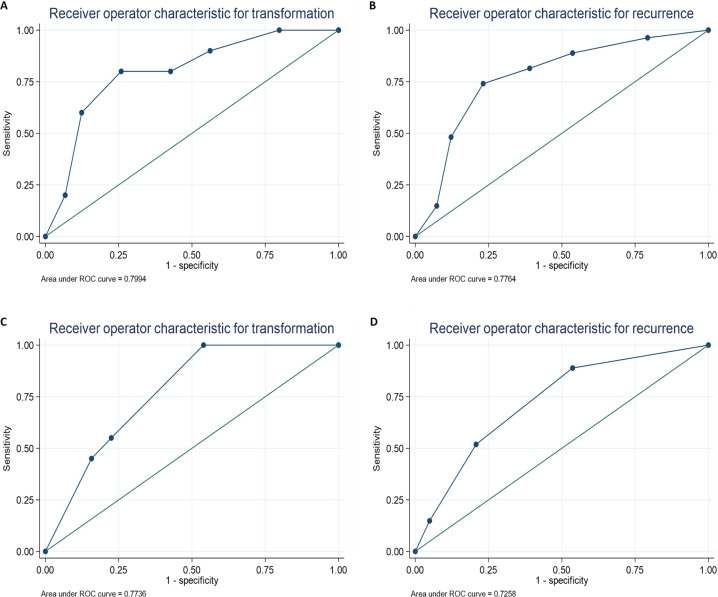
ROC curves for transformation and recurrence for feature count based on the six-point scoring system (**A**, **B**) and the two-point scoring system (**C**, **D**).

Few transformations and recurrences occurred more than five years post-baseline, and for simplicity the prognostic performance was assessed on the basis of whether the event happened rather than the time taken to occur. Figure 2 (see Supplementary Material) shows the receiver-operator characteristic curve (ROC) for these. The sensitivity and specificity appeared best balanced by using a cut off for either 4 or 5 points, with less events (for transformation and recurrence) when fewer features were present. The AUROCs for transformation and recurrence were 0.799 and 0.776, respectively.

#### Prognostic model 2: Reduced two-point scoring system

The second scoring system selected two features with the best inter-rater agreement, and which were also associated with transformation and recurrence (i.e. loss of epithelial cohesion and bulbous/drop shaped rete pegs). Figure 1C and D (see Supplementary Material) show Kaplan–Meier survival curves for time to transformation and recurrence based on the presence or absence of these two features. The combined presence of both features appeared to be associated with a higher risk of malignant transformation (39%, 95% CI 23–62%) at five years, in comparison to the presence of a single feature alone (loss of epithelial cohesion [16%, 95% CI 8–33%], bulbous/drop-shaped rete pegs [25%, 95% CI 7–69%]). However, the presence of bulbous/drop shaped rete pegs showed a higher risk of recurrence at five years (50%, 95% CI 23–85%) as compared to the presence of loss of epithelial cohesion (22%, 95% CI 11–39%) or when both of features were present in combination (43%, 95% CI 26–66%).

### Effect of patient/clinical characteristics on prognostic models

The association between patient characteristics (age, gender, intraoral site), OED histological grade and clinical outcomes were also assessed. Overall, there was a modest association between patient characteristics and clinical outcomes. However, there was a trend for higher rates of transformation and recurrence amongst older patients compared to younger, and generally with higher graded lesions as well. Moderate OED lesions were associated with a marginally higher rate of malignancy and recurrence in comparison to severe OED lesions (31% vs 15%, 38% vs 26%, respectively, Table 5). The rates for intraoral clinical sites were, at best, modestly associated with dysplasia outcomes. None of the features had an AUROC as high as that achieved by the two scoring systems.

**Table 5:** Incidence for transformation and recurrence by patient characteristics and OED histological grade.

Model	*N*	Transformation	Recurrence
Age			
<55	23	3 (13%)	3 (13%)
55–64	20	4 (20%)	5 (25%)
65–74	31	5 (16%)	6 (19%)
>=75	35	8 (23%)	13 (37%)
* AUROC*		0.526	0.591
Gender			
Female	42	8 (19%)	10 (24%)
Male	67	12 (18%)	17 (25%)
* AUROC*		0.509	0.510
WHO grade			
Mild	34	1 (3%)	2 (6%)
Moderate	48	15 (31%)	18 (38%)
Severe	27	4 (15%)	7 (26%)
* AUROC*		0.601	0.624
Binary grade			
Low	73	8 (11%)	12 (16%)
High	36	12 (33%)	15 (42%)
* AUROC*		0.665	0.650
Site of disease			
Tongue	44	10 (23%)	14 (32%)
Floor of mouth	23	3 (13%)	3 (13%)
Buccal mucosa	17	3 (18%)	5 (29%)
Gingivae	7	3 (43%)	4 (57%)
Hard palate	6	0	0
Lower lip	6	1 (17%)	1 (17%)
Soft palate	6	0	0
* AUROC*		0.544	0.547

Table 6 illustrates the effect of adding the clinical characteristics (age, gender) and histological grade (WHO and binary) to each of the prognostic models, as represented by the AUROC. Adding age and gender into the models only marginally improved the predictive ability of the scoring (6-point model: 0.810 transformation, 0.804 recurrence; 2-point model: 0.810 transformation, 0.759 recurrence), reflecting the modest association of these characteristics with transformation and recurrence. Adding the histological grade improved the models further, particularly with the WHO grade compared to the binary grade (6-point model: 0.837 vs 0.812 for transformation, 0.812 vs 0.790 for recurrence; 2-point system: 0.843 vs 0.805 for transformation, 0.780 vs 0.755 for recurrence). The number of intraoral site categories and the relatively sparse number of patients for some sites meant it was not possible to jointly model this along with the proposed scoring approaches.

**Table 6:** AUROC for each model incorporating age, gender and grading.

Model	Transformation	Recurrence
6-point score only	0.799	0.776
6-point score + age + gender	0.810	0.804
6-point score + WHO grade	0.837	0.800
6-point score + binary grade	0.812	0.790
2-point score only	0.774	0.726
2-point score + age + gender	0.810	0.759
2-point score + WHO grade	0.843	0.780
2-point score + binary grade	0.805	0.755

### Comparison of proposed models to existing grading systems

The prognostic ability of the two proposed models were compared against the existing grading systems^20^. Both the ‘six-point’ and ‘two-point’ proposed models yielded a higher AUROC than achieved by either WHO or binary grading systems, although not all these differences were statistically significant. The more detailed six-point model demonstrated a statistically significantly higher AUROC than achieved by the WHO grading system for both transformation and recurrence, but a more marginal improvement over binary grading. The two-point model showed a significant improvement over WHO grading for transformation alone (Table 7).

**Table 7:** Comparison of AUROC between two-point and six-point models with existing grading systems.

	Transformation			Recurrence		
	AUC	*p* value v WHO grade	*p* value v binary grade	AUC	*p* value v WHO grade	*p* value v binary grade
WHO grade	0.601	*-*	*-*	0.624	*-*	*-*
Binary grade	0.665	*-*	*-*	0.650	*-*	*-*
Two-point model	0.774	<0.001	0.082	0.720	0.083	0.207
Six-point model	0.799	<0.001	0.082	0.776	0.003	0.050

Finally, the prognostic performance of the new models was calculated separately for each of the three raters, reflecting how the models are likely to be used in clinical practice. Both models showed reduced prognostic ability when used by a single rater, indicating a greater risk for misclassification compared to models that were based on consensus agreement. Of the 12 single-rater AUC measures derived from the proposed models, 11 remained higher than those derived from corresponding WHO or binary grade (Table 8). Nevertheless, this analysis indicates that significant improvements on existing grading requires greater levels of agreement by assessors.

**Table 8:** AUROC for two- and six-point models calculated separately for each rater.

	AUC for Malignant Transformation				AUC for OED Recurrence			
	Rater 1	Rater 2	Rater 3	Consensus	Rater 1	Rater 2	Rater 3	Consensus
Two-point model	0.785	0.741	0.692	0.774	0.784	0.691	0.633	0.720
Six-point model	0.760	0.733	0.692	0.799	0.753	0.750	0.639	0.776

## Discussion

This study reveals important and novel information about the prognostic significance of individual histological features of OED. We have demonstrated histological feature-specific correlation of OED to malignant transformation and recurrence, which has allowed us to propose two prognostic scoring models with a potential to simplify and aid OED diagnosis and grading in the future.

Overall, nine histological features were shown to be most prevalent amongst our OED cohort (Table 2). The top two most common features were basal cell hyperplasia (crowding) and irregular surface keratin; neither of which are currently part of the WHO criteria for OED diagnosis, although our study did not show them to be strongly linked to transformation or recurrence. The least prevalent features were verrucous surface morphology, lymphocytic band, loss of epithelial cohesion, dyskeratosis and nuclear pleomorphism. Interestingly, the latter three of these features were positively associated with clinical outcomes of interest; loss of epithelial cohesion (transformation *p* = 0.003, recurrence *p* = 0.001), nuclear pleomorphism (transformation *p* = 0.005, recurrence *p* = 0.002) and dyskeratosis (recurrence *p* = 0.042) indicating that the presence of the features and not the frequency within the cohort was more important. It is evident that certain architectural features may be consistently easier to detect (even at lower magnification) as compared to other features at cellular or nuclear level. The use of immunohistochemical markers, such as Phosphorylated Histone H3 (PHH3) and Ki67 can be considered as adjuncts for the assessment of mitosis and cell proliferation^21^, although more extensive evaluation of their usefulness as a prognostic indicator in OED is needed.

Our study showed observer agreement to be the highest for verrucous surface morphology, abrupt orthokeratosis, lymphocytic band and loss of epithelial cohesion, and worst for hyperchromatism and suprabasal mitoses, further highlighting the difficulty in objective analysis of certain features in clinical practice, particularly the more ambiguously defined cytological atypia. Several studies have investigated the variability in inter- and intra-observer agreement in the diagnosis and grading of OED, with substantially different outcomes ranging from poor to high observer agreement^22–25^. One of the challenges that arises in analysing inter-rater agreement is the variation that exists in pathologists’ understanding and definitions of features due to their inherently subjective nature further complicated by the numerous changes to classifications and reporting definitions over the years. Although digital WSIs were used to mitigate the issue of variations in staining of glass slides for each pathologist, the experience of digital reporting/analysis may have caused some variation. In this study, apart from informal discussions there were no formal calibration exercises arranged prior to histological examination, as we had intended for grading and feature scoring to be most reflective of the real world and routine clinical practice. To overcome any deficiencies in feature prevalence and agreement, two chance-corrected measures were used, including bias adjusted Kappa and Gwet’s AC1, as per statistical recommendation^26^.

We found six histological features (bulbous/drop shaped rete pegs, hyperchromatism, loss of epithelial cohesion, loss of stratification, suprabasal mitoses, nuclear pleomorphism) to be associated with a greater incidence of transformation and recurrence. Although it is well acknowledged that atypical verrucous hyperplasia and/or keratoses are a subset of OPMD, and that proliferative verrucous leukoplakia has a high reported rate of malignant transformation^27,28^, we did not find a statistical association between verrucous surface morphology and clinical outcomes in our study.

Although there was a modest association between patient characteristics and clinical outcomes, there is a statistical trend for higher rates of transformation and recurrence amongst older patients as well as higher graded lesions. This trend is well supported in the literature and is thought to be related to the aggregation of genetic alterations, immunosenescence and chronic exposure to environmental risk factors with advancing age^29,30^. Interestingly though, lesions graded as moderate dysplasia were associated with a marginally higher rate of malignancy and recurrence in comparison to severe dysplasia grades (31% vs 15%, 38% vs 26%, respectively, Table 5). These findings could be explained by differences in treatments and clinical follow-up, particularly in relation to moderately graded OED lesions which are both challenging to diagnose/grade and treat. The lack of robust treatment guidelines means there is huge disparity in the management of such lesions between surgeons. Although our patient cohort was diagnosed at a single centre, differences in treatment regimens between regional hospitals, and medical/social risk factors are likely to have contributed to potential differences in their management. This further highlights the need for improved diagnostic methods which are independent of grade for more objective OED prognostication as well as more standardised treatment pathways.

We developed and assessed the potential of using two relatively simple point-based scoring systems, based on the presence or absence of certain histological features. Using the six-point model, patient scoring ‘4–6 points’ were predicted to be at the highest risk of malignant transformation and recurrence at five years, estimated at 38% (95% CI 25–55%) and 49% (95% CI 34–65%), respectively. For the two-point model, predictions suggest that the presence of bulbous/drop shaped rete pegs alone have a greater predictive association with transformation (25%, 95% CI 7–69%) and recurrence (50%, 95% CI 23–85%) at five years, compared to the presence of loss of epithelial cohesion alone (transformation at five years: 16%, 95% CI 8–33% and recurrence at five years: 22%, 95% CI 11–39%).

Comparing the two systems, the six-point model had a greater discriminant performance with more separation of the survival and ROC curves (Figs. 1 and 2, see Supplementary Material). Although it is important to highlight that based on the modest agreement between pathologists seen in this study, it is inevitable that the performance of this system may be weakened if there was only a single assessor conducting the analysis. In contrast, the two-point model is a simplified approach that focusses only on the two features with the best inter-rater agreement (presence of loss of epithelial cohesion and/or bulbous/drop shaped rete pegs which are easier to identify). This model retained predictive ability contained in the groupings (especially for transformation) whilst being less susceptible to inter-rater disagreement.

The authors acknowledge a few limitations of this study. The first relates to the relatively small sample size which was obtained from a single centre. However, the department in question is a regional and national referral centre in the UK and therefore receives tissue samples from multiple hospitals covering a wide geographical area, thereby providing a sufficiently varied cohort for this pilot study. Furthermore, whilst the sample size may be considered small, it is larger than other studies which have explored OED analysis or proposed alternate OED grading classifications^12,16,21^. Nevertheless, application of these findings to substantially larger multicentre cohorts will allow more robust validation of the proposed potential prognostic models^31^.

To the best of our knowledge, this is the first study to propose feature specific prognostic scoring models for OED. The proposed models have the potential to provide pathologists with greater insight into the risk of individual OED lesions based on feature-specific analysis, which will in turn aid clinical decision making with regards to treatment and follow-up. Larger validation of the models is required on multicentric cohorts, with prospective analysis to explore the impact of other clinical determinants such as medical/social risk factors as well as effects of treatment and frequency of monitoring. There is clearly potential for strengthening the predictive ability of the models by incorporating such measures.

Greater clarity on the definitions (and examples) for individual architectural and cytological features will greatly benefit pathologists with OED diagnosis/grading and help to improve intra-observer agreement. There is clearly a need for the development of a universal minimum dataset for the reporting of OED lesions, as well as benefit in double/consensus reporting by two pathologists to ensure accurate diagnosis and early treatment.

## Supplementary information

Supplementary Fig. S1

Supplementary Fig. S1

## Data Availability

All data generated or analysed during this study are included in this published article.

## References

[1] Lumerman H, Freedman P, Kerpel S. Oral epithelial dysplasia and the development of invasive squamous cell carcinoma. Oral Surg Oral Med Oral Pathol Oral Radiol Endod 1:321–9 (1995). 10.1016/s1079-2104(05)80226-4 7621010

[2] Warnakulasuriya S. Global epidemiology of oral and oropharyngeal cancer. Oral Oncol. 1:309–16 (2009). 10.1016/j.oraloncology.2008.06.002 18804401

[3] Bray F, Ferlay J, Soerjomataram I, Siegel RL, Torre LA, Jemal A. Global cancer statistics 2018: GLOBOCAN estimates of incidence and mortality worldwide for 36 cancers in 185 countries. CA: Cancer J. Clin 68:394–424 (2018). 10.3322/caac.21492 30207593

[4] Speight PM. Update on oral epithelial dysplasia and progression to cancer. Head Neck Pathol 1:61–6 (2007). 10.1007/s12105-007-0014-5 PMC2807503 20614284

[5] Locca O, Sollecito TP, Alawi F, Weinstein GS, Newman JG, De Virgilio A et al. Potentially malignant disorders of the oral cavity and oral dysplasia: A systematic review and meta‐analysis of malignant transformation rate by subtype. Head Neck 42:539–55 (2020). 10.1002/hed.26006 31803979

[6] Warnakulasuriya S, Kujan O, Aguirre‐Urizar JM, Bagan JV, González‐Moles MA, Kerr AR (2021). Oral potentially malignant disorders: A consensus report from an international seminar on nomenclature and classification, convened by the WHO Collaborating Centre for Oral Cancer. Oral Dis.

[7] Reibel J. Prognosis of oral pre-malignant lesions: significance of clinical, histopathological, and molecular biological characteristics. Crit Rev Oral Biol Med 14:47–62 (2003). 10.1177/154411130301400105 12764019

[8] Speight PM, Khurram SA, Kujan O (2018). Oral potentially malignant disorders: risk of progression to malignancy. Oral Surg Oral Med Oral Pathol Oral Radiol.

[9] Odell E, Kujan O, Warnakulasuriya S, Sloan P (2021). Oral epithelial dysplasia: recognition, grading and clinical significance. Oral Dis.

[10] El-Naggar AK, Chan JK, Grandis JR, Takata T, Slootweg PJ. WHO Classification of Head and Neck Tumors (4th ed. Vol. 9) Tumours of the oral cavity and mobile tongue (p 112) (IARC, 2017).

[11] Warnakulasuriya S. Histological grading of oral epithelial dysplasia: revisited. J. Pathol 194:294–7 (2001). 10.1002/1096-9896(200107)194:3<294::AID-PATH911>3.0.CO;2-Q 11439360

[12] Kujan O, Khattab A, Oliver RJ, Roberts SA, Thakker N, Sloan P. Why oral histopathology suffers inter-observer variability on grading oral epithelial dysplasia: an attempt to understand the sources of variation. Oral Oncol 1:224–31 (2007). 10.1016/j.oraloncology.2006.03.009 16931119

[13] Leemans CR, Snijders PJ, Brakenhoff RH. The molecular landscape of head and neck cancer. Nat Rev Cancer 18:269–82 (2018). 10.1038/nrc.2018.11 29497144

[14] Califano J, Van Der Riet P, Westra W, Nawroz H, Clayman G, Piantadosi S et al. Genetic progression model for head and neck cancer: implications for field cancerization. Cancer Res. 1:2488–92 (1996). 8653682

[15] Kujan O, Oliver RJ, Khattab A, Roberts SA, Thakker N, Sloan P. Evaluation of a new binary system of grading oral epithelial dysplasia for prediction of malignant transformation. Oral Oncol 1:987–93 (2006). 10.1016/j.oraloncology.2005.12.014 16731030

[16] Nankivell P, Williams H, Matthews P, Suortamo S, Snead D, McConkey C et al. The binary oral dysplasia grading system: validity testing and suggested improvement. Oral Surg Oral Med Oral Pathol Oral Radiol 1:87–94 (2013). 10.1016/j.oooo.2012.10.015 23217539

[17] Kramer IR, Lucas RB, El-Labban N, Lister L. The use of discriminant analysis for examining the histological features of oral keratoses and lichen planus. Br. J. Cancer 24:673–83 (1970). 10.1038/bjc.1970.80 PMC2008723 5503594

[18] MacDonald DG, Saka SM. Structural indicators of the high risk lesion. In: Johnson NE, editor. Risk Markers for Oral Diseases. Oral Cancer: Detection of Patients and Lesions at Risk. C.U.P. (1991).

[19] Stata Statistical Software: Release 17. College Station, TX: StataCorp LLC. StataCorp (2019).

[20] DeLong ER, DeLong DM, Clarke-Pearson DL. Comparing the areas under two or more correlated receiver operating characteristic curves: a nonparametric approach. Biometrics. 1:837–45 (1988). 3203132

[21] Sudarshini N, Banavar SR, Nambiar SK, Augustine D, Haragannavar VC, Sowmya S et al. Immunohistochemical Stain-Phosphohistone H3: Most Specific Mitotic Marker. J. clin. diagn 1;12 (2018).

[22] Pindborg JJ, Reibel J, Holmstrup P. Subjectivity in evaluating oral epithelial dysplasia, carcinoma in situ and initial carcinoma. J. Oral Pathol Med 14:698–708 (1985). 10.1111/j.1600-0714.1985.tb00549.x 3932623

[23] Karabulut A, Reibel J, Therkildsen MH, Praetorius F, Nielsen HW, Dabelsteen E. Observer variability in the histologic assessment of oral premalignant lesions. J. Oral Pathol Med 24:198–200 (1995). 10.1111/j.1600-0714.1995.tb01166.x 7616457

[24] DJ, Brothwell, Lewis DW, Leong I, Jordan RCK, and Leake J. L. Observer agreement in the grading of oral epithelial dysplasia. Community Dent. Oral Epidemiol 31:300–5 (2003). 10.1034/j.1600-0528.2003.00013.x 12846853

[25] Fischer DJ, Epstein JB, Morton Jr TH, Schwartz SM. Interobserver reliability in the histopathologic diagnosis of oral pre‐malignant and malignant lesions. J. Oral Pathol Med 33:65–70 (2004). 10.1111/j.1600-0714.2004.0037n.x 14720191

[26] Flight L, Julious SA. The disagreeable behaviour of the kappa statistic. Pharm. Stat 14:74–8 (2015). 10.1002/pst.1659 25470361

[27] Müller S. Oral epithelial dysplasia, atypical verrucous lesions and oral potentially malignant disorders: focus on histopathology. Oral Surg Oral Med Oral Pathol Oral Radiol 1:591–602 (2018). 10.1016/j.oooo.2018.02.012 29606637

[28] Thompson LD, Fitzpatrick SG, Müller S, Eisenberg E, Upadhyaya JD, Lingen MW et al. Proliferative verrucous leukoplakia: an expert consensus guideline for standardized assessment and reporting. Head Neck Pathol 15:572–87 (2021). 10.1007/s12105-020-01262-9 PMC8134585 33415517

[29] Gupta PC, Mehta FS, Daftary DK, Pindborg JJ, Bhonsle RB, Jalnawalla PN et al. Incidence rates of oral cancer and natural history of oral precancerous lesions in a 10-year follow-up study of Indian villagers. Community Dent. Oral Epidemiol. 8:283–333 (1980). 10.1111/j.1600-0528.1980.tb01302.x 6937277

[30] Ranganathan K, Kavitha L. Oral epithelial dysplasia: Classifications and clinical relevance in risk assessment of oral potentially malignant disorders. J Oral Maxillofac Pathol 23:19 (2019). 10.4103/jomfp.JOMFP_13_19 PMC6503768 31110412

[31] Moons KG, Royston P, Vergouwe Y, Grobbee DE, Altman DG. Prognosis and prognostic research: what, why, and how? BMJ 23;338 (2009). 10.1136/bmj.b375 19237405

