# Supplementary figures and images for: Prediction of malignant transformation and recurrence of oral epithelial dysplasia using architectural and cytological feature specific prognostic models
Source: Mod Pathol. 2022 Mar 31;35(9):1151–9. doi: 10.1038/s41379-022-01067-x (PMC9424112; doi:10.1038/s41379-022-01067-x)

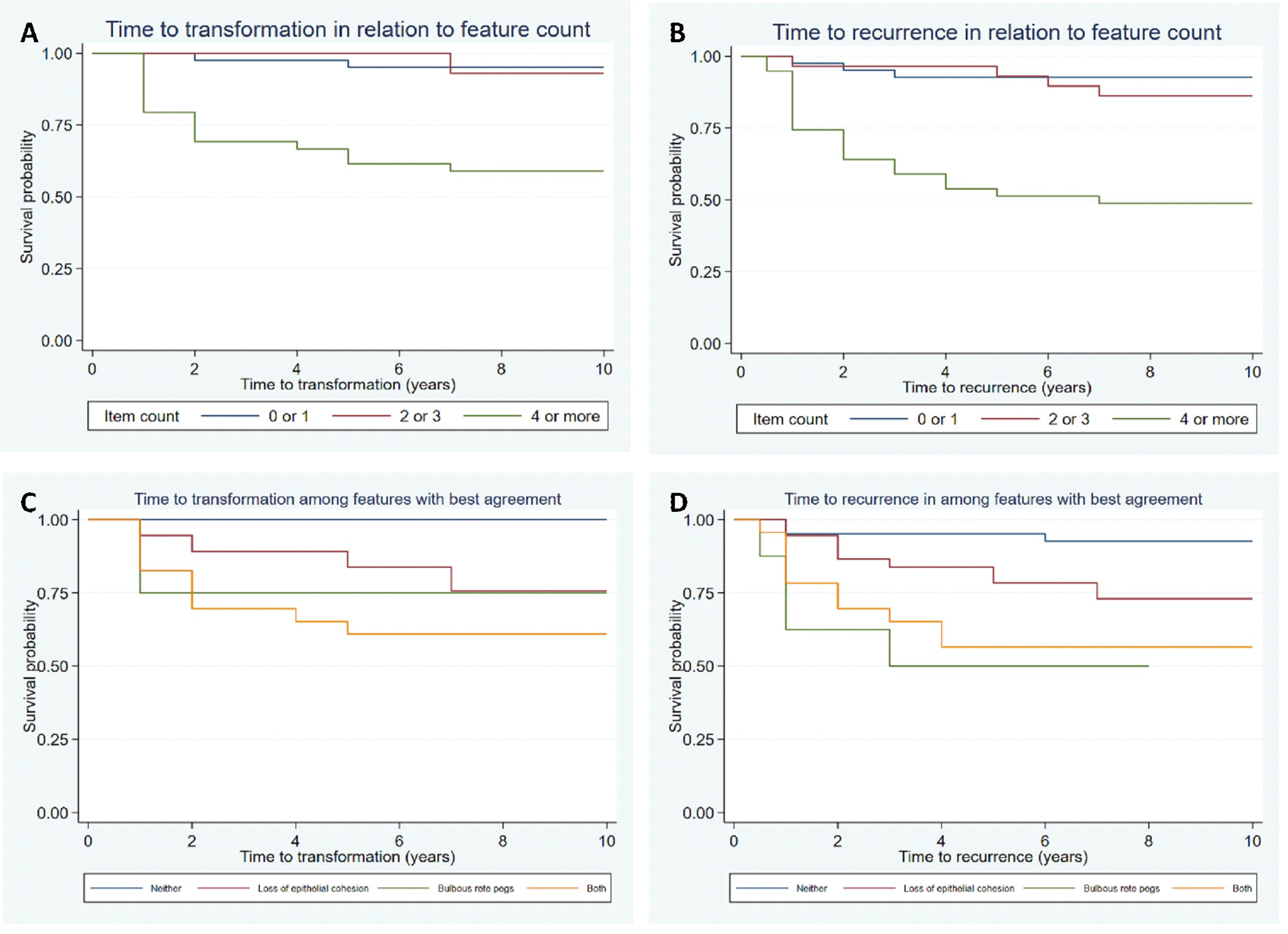

Supplement: Supplementary file 1 — Supplementary Fig. S1 [file 41379_2022_1067_MOESM1_ESM.jpg]

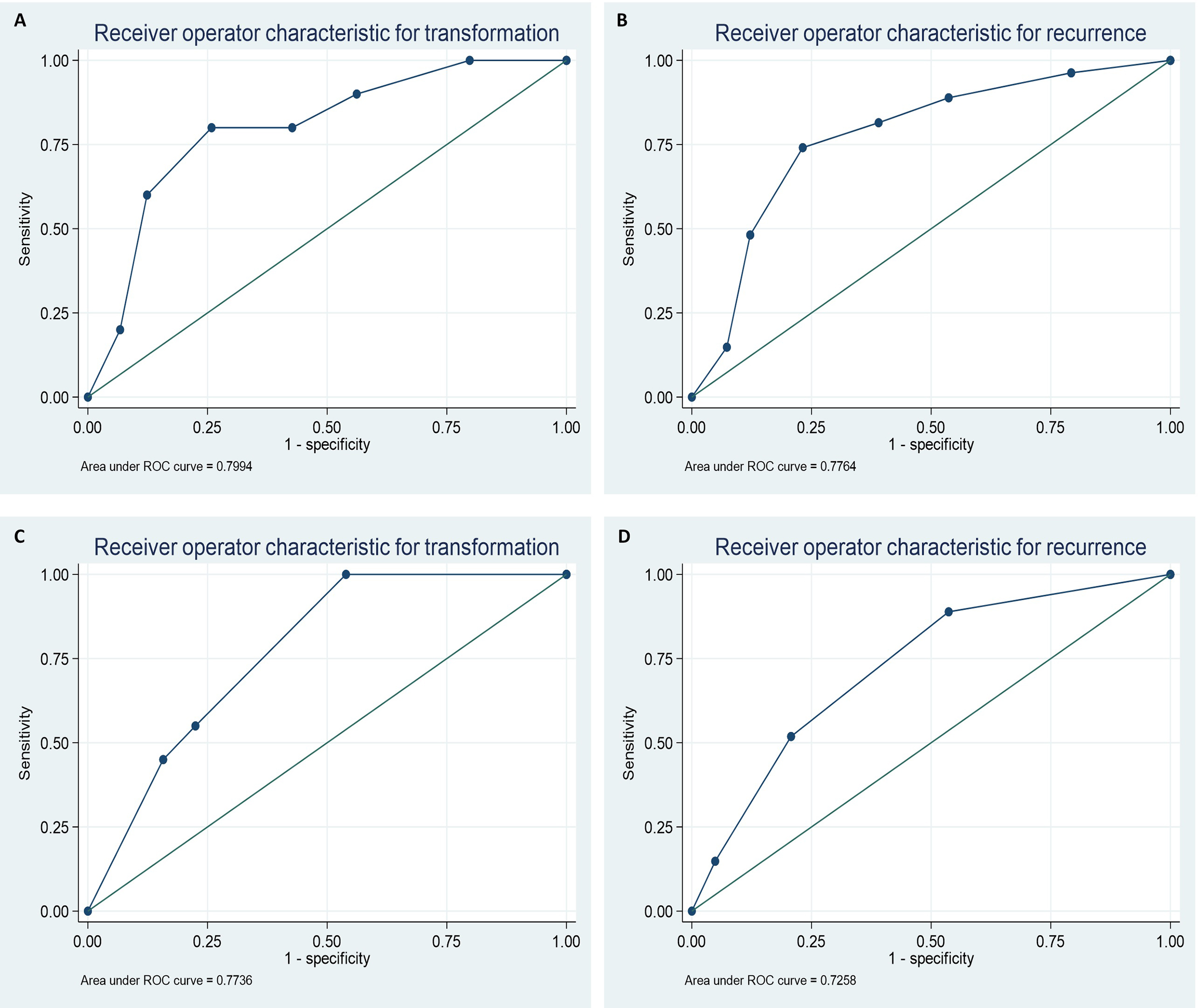

Supplement: Supplementary file 2 — Supplementary Fig. S1 [file 41379_2022_1067_MOESM2_ESM.jpg]
